# Peripheral microvascular function is linked to cardiac involvement on cardiovascular magnetic resonance in systemic sclerosis–related pulmonary arterial hypertension

**DOI:** 10.1093/ehjci/jeae001

**Published:** 2024-01-03

**Authors:** Jacqueline L Vos, Jacqueline M J Lemmers, Saloua El Messaoudi, Miranda Snoeren, Arie P J van Dijk, Anthonie L Duijnhouwer, Laura Rodwell, Sander I van Leuven, Martijn C Post, Madelon C Vonk, Robin Nijveldt

**Affiliations:** Department of Cardiology, Radboud University Medical Center, Geert Grooteplein 10, 6525 GA, Nijmegen, The Netherlands; Department of Rheumatology, Radboud University Medical Center, Nijmegen, The Netherlands; Department of Cardiology, Radboud University Medical Center, Geert Grooteplein 10, 6525 GA, Nijmegen, The Netherlands; Department of Radiology, Radboud University Medical Center, Nijmegen, The Netherlands; Department of Cardiology, Radboud University Medical Center, Geert Grooteplein 10, 6525 GA, Nijmegen, The Netherlands; Department of Cardiology, Radboud University Medical Center, Geert Grooteplein 10, 6525 GA, Nijmegen, The Netherlands; Department of Health Evidence, Section Biostatistics, Radboud University Medical Center, Nijmegen, The Netherlands; Department of Rheumatology, Radboud University Medical Center, Nijmegen, The Netherlands; Department of Cardiology, St. Antonius Hospital, Nieuwegein, The Netherlands; Department of Cardiology, University Medical Center Utrecht, Utrecht, The Netherlands; Department of Rheumatology, Radboud University Medical Center, Nijmegen, The Netherlands; Department of Cardiology, Radboud University Medical Center, Geert Grooteplein 10, 6525 GA, Nijmegen, The Netherlands

**Keywords:** systemic sclerosis, pulmonary arterial hypertension, cardiac magnetic resonance imaging, parametric mapping, nailfold capillaroscopy

## Abstract

**Aims:**

Systemic sclerosis (SSc) is characterized by vasculopathy, inflammation, and fibrosis, and carries one of the worst prognoses if patients also develop pulmonary arterial hypertension (PAH). Although PAH is a known prognosticator, patients with SSc–PAH demonstrate disproportionately high mortality, presumably due to cardiac involvement. In this cross-sectional study, the relationship between cardiac involvement revealed by cardiovascular magnetic resonance (CMR) and systemic microvascular disease severity measured with nailfold capillaromicroscopy (NCM) in patients with SSc–PAH is evaluated and compared with patients with idiopathic PAH (IPAH).

**Methods and results:**

Patients with SSc–PAH and IPAH underwent CMR, echocardiography, and NCM with post-occlusive reactivity hyperaemia (PORH) testing on the same day. CMR imaging included T_2_ (oedema), native, and post-contrast T_1_ mapping to measure the extracellular volume fraction (ECV, fibrosis) and adenosine-stress-perfusion imaging measuring the relative myocardial upslope (microvascular coronary perfusion). Measures of peripheral microvascular function were related to CMR indices of oedema, fibrosis, and myocardial perfusion. SSc-PAH patients (*n* = 20) had higher T_2_ values and a trend towards a higher ECV, compared with IPAH patients (*n* = 5), and a lower nailfold capillary density (NCD) and reduced capillary recruitment after PORH. NCD correlated with ECV and T_2_ (*r* = −0.443 and −0.464, respectively, *P* < 0.05 for both) and with markers of diastolic dysfunction on echocardiography. PORH testing, but not NCD, correlated with the relative myocardial upslope (*r* = 0.421, *P* < 0.05).

**Conclusion:**

SSc-PAH patients showed higher markers of cardiac fibrosis and inflammation, compared with IPAH patients. These markers correlated well with peripheral microvascular dysfunction, suggesting that SSc-driven inflammation and vasculopathy concurrently affect peripheral microcirculation and the heart. This may contribute to the disproportionate high mortality in SSc–PAH.

## Graphical Abstract

**Graphical Abstract:**
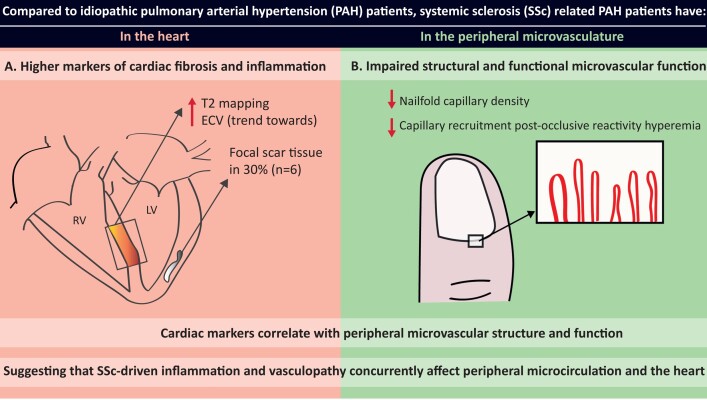

## Introduction

Systemic sclerosis (SSc) is a systemic immune-mediated disease that is characterized by vasculopathy, inflammation, and fibrosis of skin and internal organs.^1^ Pulmonary arterial hypertension (PAH) is a common complication, affecting around 12% of patients with SSc.^2^ Pulmonary vascular disease increases pulmonary vascular resistance, leading to PAH and to elevate right ventricular (RV) afterload and hence induces RV remodelling with increased contractility and hypertrophy.^3^ This so-called RV adaptation ultimately determines patient prognosis.^3^ Compared with other patients with PAH, SSc–PAH patients have the worst prognosis, exposing a disproportionately high mortality.^2^ It is suggested that intrinsic myocardial dysfunction^4^ due to primary cardiac involvement, such as coronary microvascular dysfunction, inflammation, and fibrosis, is the leading cause of worse outcome in patients with SSc–PAH.^5–8^

Clinical assessment of peripheral vasculopathy in patients with SSc includes the evaluation of the microvascular bed by means of nailfold capillaromicroscopy (NCM). In SSc, this may be severely affected, and specific alterations are currently used as a classification criterion in SSc^1,9^ and are also considered to reflect internal organ involvement in SSc.^10^ The peripheral capillaries are thought to mirror systemic vasculopathy, and NCM with post-occlusive reactivity hyperaemia (PORH) testing of the middle phalanx is known to be altered in patients with coronary artery disease.^11^

Cardiovascular magnetic resonance (CMR) imaging is the recommended imaging modality to investigate the myocardial involvement of systemic inflammatory diseases.^12^ It combines functional assessment of both ventricles and the atria by volumetric analyses and strain imaging on the one hand, and tissue characterization to determine oedema or fibrosis with parametric mapping and late gadolinium enhancement (LGE) on the other.^13,14^ Additionally, with the use of a pharmacological stress agent, myocardial perfusion can be measured to detect epicardial coronary artery disease as well as microvascular dysfunction.^15,16^ It is, therefore, the ideal technique to investigate the incremental effect of systemic inflammation on myocardial involvement in SSc–PAH compared with patients with isolated PAH, to reach a better understanding of the mechanism leading to a worse outcome in patients with SSc–PAH.

The aim of the current study is to investigate the relationship between cardiac involvement as revealed by CMR and systemic vasculopathy severity as measured with NCM in patients with SSc–PAH compared with patients with isolated PAH [heritable or idiopathic PAH (IPAH)].

## Methods

### Study population

In this cross-sectional pilot study, 20 patients with SSc–PAH and 5 patients with idiopathic or familial PAH (IPAH) were included. PAH was defined as a mean pulmonary arterial pressure ≥25 mmHg on cardiac catheterization with a pulmonary capillary wedge pressure of ≤15 mmHg, or a combination of PAH with pulmonary hypertension (PH) due to left-sided heart disease [World Health Organization (WHO) Group II] or interstitial lung disease (ILD, WHO Group III), according to the ESC/ERS 2015 guidelines.^17^ Patients with SSc fulfilled the 2013 American College of Rheumatology/European League against Rheumatism classification criteria.^1^ Exclusion criteria were: (i) a history of myocardial infarction or ischaemic heart failure, and/or moderate-to-severe left-sided valve stenosis or regurgitations and (ii) known contra-indications for CMR or adenosine stress testing (e.g. severe claustrophobia, metal implants, known glomerular filtration rate <30 mL/min).

For all patients, the following examinations were performed on the same day: CMR, transthoracic echocardiography (TTE), blood sampling, a 6 min walking distance (6MWD) test, and NCM with PORH testing. In addition, a pulmonary function test was performed if no pulmonary function test was available within 3 months of the study visit. TTE parameters include the peak early filling (*E*-wave) and late diastolic filling (*A*-wave) velocities, and the early diastolic velocities at the lateral mitral annulus (*e*ʹ lateral), to calculate *E*/*A* ratios and *E*/*e*ʹ lateral. In addition, tricuspid annular plane systolic excursion was measured and RV systolic pressure (RVSP) was calculated (for details, see Supplementary data online, *Methods*). The Ethical Review boards approved the study. Written informed consent was obtained from all study participants prior to inclusion.

### Peripheral microvascular evaluation

The images were analysed according to the EULAR Study Group on Microcirculation in Rheumatic Diseases/Scleroderma Clinical Trials Consortium on Capillaroscopy^9^ by two experienced physicians, and consensus was reached in all cases (M.C.V. and J.M.J.L.). A normal capillary density is defined as ≥7 capillaries/mm. Besides this capillary density, the patterns were evaluated as normal, non-specific changes, early SSc pattern, active SSc pattern, or late SSc pattern.^18^

The PORH images were taken from the dorsal skin of the middle phalanx of the third finger. The images were acquired at rest, directly after arterial occlusion and during venous congestion (achieved by inflating blood pressure cuff >60 mmHg above systolic blood pressure, and to 60 mmHg, respectively).^11,19^ All capillaries within 1 mm^2^ were counted by two investigators. The capillaries at rest represent the functional capillary density. The capillaries at venous congestion represent the structural number of capillaries, including the non-perfused capillaries (not visible at rest). Temporary arterial occlusion creates reactive hyperaemia by releasing endothelial mediators, resulting in vasodilation, and therefore, the total number of capillaries after PORH represents both functional and structural changes.^11,19^ For more detailed acquisition and analysis, see Supplementary data online, *Methods*.

### CMR acquisition

Detailed CMR acquisition and analysis protocols are described in Supplementary data online, *Methods*. All patients were scanned on a commercially available clinical CMR scanner (1.5 T Siemens Avanto). Cine and LGE images were made using short-axis orientation [covering the entire left ventricular (LV)] and long-axis orientation (two, three, and four chambers). T_2_ mapping was acquired using a bright-blood T_2_ prepared, steady-state free precession sequence. Native and post-contrast T_1_ mapping images were acquired using the Shortened Modified Look-Locker Inversion Recovery sequence. Haematocrit, to calculate the extracellular volume fraction (ECV), was measured on the same day.^14^ Stress first-pass perfusion imaging was performed with a gadolinium-based contrast agent to measure the relative myocardial upslope and to evaluate regional perfusion defects to exclude significant epicardial coronary stenoses. Ten to 15 min after contrast injection, LGE images were acquired using a 2D, segmented inversion-recovery-prepared gradient echo pulse sequence.

### CMR analysis

All post-processing analyses were performed using Medis Qstrain software (Medis Medical Imaging Systems, version 2.0.48.8, The Netherlands). LV and RV volumes and mass were measured on the short-axis cine images, and ejection fraction (EF) was calculated. Left atrial (LA) reservoir strain was the average strain measured on the two- and four-chamber long-axis cine images, and LV global longitudinal strain (GLS) was the average strain measured on the three long-axis cine images. Right atrial (RA) reservoir strain and RV GLS were measured on the four-chamber long-axis cine images. According to the CMR-based cardiovascular phenotypes associated with distinct outcomes published in the recent study of Knight *et al*.,^20^ SSc–PAH patients were clustered into the following groups: ‘RV failure’, ‘biventricular failure’, ‘normal function, large cavity’, ‘normal function, small cavity’, and ‘normal function, average cavity’.

#### Parametric mapping

The analysis was performed in accordance with the recommendations of the Society of Cardiovascular Magnetic Resonance, wherein diffuse cardiac disease was assessed by manually delineating a single region of interest on the septal mid-ventricular short-axis images.^21^ Additionally, the entire myocardium (excluding regions with focal LGE) was delineated using endocardial and epicardial contours on all three short-axis and four-chamber long-axis images.^21^ The ECV was used to evaluate cardiac fibrosis. T_2_ mapping was used to evaluate cardiac oedema. T_2_ ≥ 55 ms was defined as a sign of myocardial oedema.

#### Microvascular coronary perfusion

The semiquantitative analysis of myocardial perfusion during stress was calculated by measuring the mean relative myocardial upslope of the signal intensity time curve of the contrast agent. LV endocardial and epicardial contours were drawn on the three short-axis first-pass perfusion images, as described previously,^22^ and the mean relative myocardial upslope was automatically calculated.

### Statistical analysis

Variables are displayed as numbers (percentage) or median (interquartile range). Linear correlations between continuous variables were assessed using the Pearson’s correlation coefficient. To assess differences between patients with SSc–PAH and IPAH, the Mann–Whitney *U* test for continuous variables and the Fisher’s exact test for categorical variables were used. *Post hoc* testing to account for false discovery was performed by employing the Benjamini–Hochberg false-discovery correction method (using a false-discovery rate of 10%). Statistical analysis was performed using SPSS 26.0 (IBM Corp., Armonk, NY, USA) software. A two-tailed *P*-value <0.05 was considered statistically significant.

## Results

All 20 patients with SSc–PAH and 5 patients with IPAH were diagnosed with PAH (WHO Group I), as determined on right heart catheterization. In four patients with SSc–PAH, there was a combined diagnosis with another PH WHO Group: one with left-sided heart disease (WHO Group II) and three with ILD (WHO Group 3). Clinical characteristics are displayed in *Table 1*. Patients with SSc–PAH and IPAH were comparable in age and sex. The median PAH duration was 4 (2–7) years in patients with SSc–PAH and 7 (4–11) years in patients with IPAH (*P* = 0.163). *Table 1* displays the current drug treatment. Four IPAH patients (80%) and six SSc–PAH patients (30%) received triple therapy, comprising an endothelin receptor antagonist, prostacyclin IP-receptor-agonist, and a phosphodiesterase Type 5 inhibitor in most. Ten SSc–PAH patients (50%) and the remaining IPAH patient received dual therapy, and the predominant combination was an endothelin receptor antagonist combined with a phosphodiesterase type 5 inhibitor (73%) or a guanylate cyclase stimulator (27%). Six patients with SSc–PAH (32%) received oxygen therapy. Treatment history revealed that four patients had a prostacyclin analogue prior to inclusion, which was switched to a prostacyclin IP-receptor agonist in two patients (one patient with SSc–PAH and one patient with IPAH), and was discontinued in two patients with SSc–PAH due to side-effects. In three patients, the phosphodiesterase type 5 inhibitors were replaced with a prostacyclin IP-receptor-agonist prior to inclusion, in two patients due to side-effects. Sixty per cent of patients with SSc–PAH had a WHO functional class of ≥ III compared with none of the patients with IPAH, and there was a trend towards a lower 6MWD (*P* = 0.098). In addition, patients with SSc–PAH had a worse mean diffusing capacity of the lungs for carbon monoxide (DLCO) of predicted (38 vs. 71%, *P* = 0.001) and a significantly higher N-terminal pro B-type natriuretic peptide (NT-proBNP).

**Table 1:** Baseline characteristics

	SSc–PAH (*n* = 20)	IPAH (*n* = 5)	*P*-value
Age (years)	71 (62–77)	69 (47–77)	0.562
Male (*n*)	3 (15%)	1 (20%)	1.00
BSA (m^2^)	1.9 (1.7–2.0)	1.8 (1.7–2.0)	0.909
Comorbidities			
Hypertension (*n*)	10 (50%)	1 (20%)	0.341
Hypercholesterolaemia (*n*)	2 (10%)	2 (40%)	0.166
Diabetes mellitus (*n*)	1 (5%)	0	1.00
Systemic sclerosis characteristics			
Diffuse systemic sclerosis (*n*)	4 (20%)		
Raynaud phenomenon (*n*)	20 (100%)		
Raynaud’s phenomenon duration (years)	15 (8–28)		
Non-Raynaud’s phenomenon duration (years)	11 (6–16)		
Modified Rodnan Skin Score (points)	3 (1–6)		
Arthritis (*n*)	4 (20%)		
Interstitial lung disease (*n*)	15 (75%)		
Pulmonary hypertension characteristics			
WHO category			
WHO Type I	16 (80%)	5 (100%)	
WHO Type I + II	1 (5%)		
WHO Type I + III	3 (15%)		
Duration of PH (years)	4 (2–7)	7 (4–11)	0.163
Treatment of PH (*n*)			
None	1 (5%)	0	
Monotherapy	3 (15%)	0	
Dual therapy	10 (50%)	1 (20)	
Triple therapy	6 (30%)	4 (80)	
Type of drug			
Endothelin receptor antagonists	17 (85%)	5 (100%)	0.496
Phosphodiesterase Type 5 inhibitors	14 (70%)	3 (60%)	0.525
Guanylate cyclase stimulators	3 (15%)	2 (40%)	0.252
Prostacyclin analogue	1 (5%)	0 (0%)	0.800
Prostacyclin IP-receptor-agonist	6 (30%)	4 (80%)	0.064
O_2_ therapy (*n*)	6 (32%)	0 (0%)	0.280
Functional status assessment			
WHO functional Class ≥ III (*n*)	12 (60%)	0 (0%)	0.039^a^
6 min walking distance (m)	354 (279–450)	432 (413–491)	0.098
Pulmonary function testing			
Forced vital capacity (% of predicted)	88 (77–107)	98 (85–129)	0.210
DLCO (% of predicted)	38 (30–52)	71 (59–88)	**0**.**001**
Laboratory testing			
NT-proBNP (pg/mL)	355 (150–1450)	100 (57–135)	**0**.**003**
High-sensitive troponin T (ng/L)	14 (8–19)	14 (5–22)	0.436

Values are in medians (interquartile range) or number (%). *P-*values in bold indicate statistically significant correlations (*P* < 0.05).

BSA, body surface area; DLCO, diffusing capacity of the lungs for carbon monoxide; (I)PAH, idiopathic pulmonary arterial hypertension; SSc, systemic sclerosis; NT-proBNP, N-terminal pro-brain natriuretic peptide.

^a^Non-significant difference after *post hoc* testing.

The RVSP did not differ significantly between patients with SSc–PAH and IPAH (*Table 2*). Furthermore, echocardiographic assessment revealed lower median *e*ʹ lateral velocities and subsequent trend towards higher *E*/*e*ʹ ratios in patients with SSc–PAH compared with patients with IPAH.

**Table 2:** Baseline echocardiographic and CMR characteristics

	SSc–PH (*n* = 20)	IPAH (*n* = 5)	*P*-value
Echocardiography			
*E* velocity (m/s)	0.7 (0.6–0.8)	0.6 (0.5–0.7)	0.294
*e*ʹ lateral velocity (m/s)	0.08 (0.07–0.10)	0.12 (0.09–0.15)	**0**.**023**
*E*/*A* ratio	0.7 (0.7–1.0)	0.8 (0.5–1.0)	0.818
*E*/*e*ʹ ratio	7.8 (6.0–10.7)	5.6 (3.7–6.7)	0.030^a^
RVSP (mmHg)	57 (35–63)	69 (56–77)	0.123
Cardiovascular magnetic resonance imaging			
LV EDVi (mL/m^2^)	73 (66–82)	68 (64–75)	0.447
LV EF (%)	63 (58–69)	60 (59–64)	0.587
LV longitudinal strain (%)	−22 (−20 to −25)	−24 (−22 to −27)	0.336
Indexed LV mass (g/m^2^)	47 (44–53)	35 (33–39)	**0**.**002**
LA reservoir strain (%)	29 (26–33)	37 (31–40)	0.067
RV EDVi (mL/m^2^)	83 (69–107)	91 (76–104)	0.621
RV EF (%)	54 (46–58)	48 (44–54)	0.371
RV longitudinal strain (%)	−24 (−21 to −27)	−24 (−22 to −26)	0.965
Indexed RV mass (g/m^2^)	12 (10–16)	12 (12–17)	0.621
RA reservoir strain (%)	36 (22–41)	34 (28–42)	0.892
Tissue characterization and coronary perfusion			
Native T_1_ value (ms)^b^	966 (932–985)	899 (891–921)	**0**.**004**
ECV (%)^b^	30 (28–34)	28 (24–29)	0.060
T_2_ values (ms)^c^	50 (48–54)	45 (42–46)	**0**.**001**
Relative myocardial upslope (%)	16 (13–19)	17 (16–20)	0.336
LGE presence (non-insertion RV)	6 (30%)	0	0.289
Insertion RV	9 (45%)	5 (100%)	**0**.**046**
Ischaemic	1 (5%)	0	1.00
Non-ischaemic	5 (25%)	0	0.544

Values are in medians (interquartile range) or number (%). The *P-*values in bold indicate statistically significant correlations (*P* < 0.05). Abbreviations are as in *Table 1*.

CMR, cardiovascular magnetic resonance; ECV, extracellular volume fraction; EDVi, end-diastolic volume indexed; EF, ejection fraction; LGE, late gadolinium enhancement; LV, left ventricular; RV, right ventricular; RVSP, RV systolic pressure.

^a^Non-significant difference after *post hoc* testing.

^b^T_1_ values and ECVs are measured outside the areas of LGE.

^c^T_2_ map missing in one IPAH patient.

### Cardiac function and tissue characterization

Due to technical reasons, T_2_ mapping could not be performed in one patient with IPAH, and another patient with SSc–PAH did not undergo adenosine-stress imaging due to shortness of breath. There were no statistical differences between the LV and the RV volumes, global function, or strain parameters. The indexed LV mass was slightly higher in SSc–PAH than in IPAH patients (*Table 2*).

Patients with SSc–PAH had both a higher native T_1_, T_2_ (cardiac oedema) and a trend towards a higher ECV (cardiac fibrosis, *P* = 0.06) compared with patients with IPAH (*Table 2*). An analysis of the entire myocardium revealed similar findings for T_1_ and T_2_ values, while revealing a significantly higher ECV in patients with SSc–PAH compared with patients with IPAH as well (*P* < 0.01 for all; Supplementary data online, *Table S1*). Five patients with SSc–PAH (25%) showed signs of myocardial oedema (T_2_ ≥ 55 ms), whereas none of the patients with IPAH did. Patients with SSc–PAH with myocardial oedema had a higher ECV [37 (30–38) vs. 29 (27–32)%, *P* = 0.037], NT-proBNP [1600 (970–3600) vs. 210 (120–570) ng/L, *P* = 0.006], and estimated RVSP [63 (53–87) vs. 38 (33–61) mmHg, *P* = 0.043] and a worse DLCO [35 (20–37) vs. 44 (33–55)%, *P* = 0.039] than patients with SSc–PAH without oedema (*n* = 15). There were no significant differences in cardiac systolic or diastolic functional parameters (data not provided).

Five patients with SSc–PAH (25%) had non-ischaemic LGE (epi- and mid-myocardial in four patients, and in one patient, a focal subendocardial spot in the apex). One patient with SSc–PAH had subendocardial (and transmural) LGE in the inferolateral basal region, which could be attributable to a previous (unknown) myocardial infarction of the left circumflex artery. All patients with IPAH had LGE at the RV insertion points (hinge point fibrosis), compared with nine patients with SSc–PAH (45%, *P* = 0.046). One patient with SSc–PAH had a chronic total occlusion of the right coronary artery with a concomitant stress-perfusion defect, and these segments were not included in the stress-perfusion image analysis. With respect to myocardial perfusion and coronary microvascular function, there was no statistical difference between patients with SSc–PAH and IPAH in the relative myocardial upslope. T_2_ values significantly correlated with LV diastolic functional parameters, CMR-derived LA reservoir strain, and echocardiographic *e*ʹ lateral velocity and *E*/*e*ʹ ratio (*Table 3*). ECV and T_2_ correlated with NT-proBNP and the DLCO of predicted. T_2_ values and the relative myocardial upslope correlated with the 6MWD, a functional status marker. There were no significant correlations between CMR tissue characterization and RVSP (*Table 3*), and systolic LV and RV function as determined by EF or strain (data not provided).

**Table 3:** Correlations of cardiac tissue characteristics with cardiac function and disease severity markers

	Indexed LV mass (g/m^2^)	LA reservoir strain (%)	*E*′ lateral velocity	*E*/*e*′ ratio	Estimated RVSP (mmHg)	NT-proBNP (pg/mL)	6MWD of predicted (%)	DLCO of predicted (%)
ECV (%)	0.129	−0.311	−0.296	0.165	0.238	**0.517**	−0.396	**−0**.**529**
T_2_ (ms)	**0**.**513**	**−0**.**518**	−0.375	**0**.**450**	0.101	**0**.**481**	**−0**.**619**	**−0**.**555**
Relative myocardial upslope (%)	−0.325	0.351	0.249	**−0**.**456**	−0.144	**−0**.**527**	**0**.**447**	0.300

The *P*-values in bold indicate statistically significant correlations (*P* < 0.05). Abbreviations are as in previous tables.

Using the CMR-based cardiovascular phenotypes by Knight *et al*.,^20^ nine patients (45%) fell into the ‘normal function, average cavity’ category. They had a median ECV of 29 (27–30)% and T_2_ of 49 (48–51) ms. Two patients (10%) were categorized as ‘normal function, small cavity’, one patient had normal values of the parametric mapping (ECV 26%, T_2_ 50 ms), the other had elevated parametric mapping values (ECV 32%, T_2_ 55 ms). Two patients (20%) fell into the ‘RV failure’ category with elevated parametric mapping values (ECV 39 and 35%, and T_2_ 59 and 53 ms, respectively). Seven patients (35%) were categorized as ‘normal function, large cavity’, with a high median ECV of 33 (28–37)%, and T_2_ values of 50 (49–55) ms. In terms of the cluster-based relation to outcome as evaluated by Knight *et al.*,^20^ 11 patients (55%) fell into one of the clusters associated with better prognosis (‘normal function, average cavity’ or ‘normal function, small cavity’), and 9 patients (45%) in one of the clusters associated with worse prognosis (‘RV failure’ and ‘normal function, large cavity’). Interestingly, compared with the patients in clusters associated with good prognosis, patients in the clusters associated with worse prognosis had a significantly higher ECV (*P* = 0.016). There were no significant differences in T_2_ values, presence of LGE, age, duration of PAH, WHO functional class, DLCO, and 6MWD.

### Peripheral microvascular anatomy and function

**Figure 1:**
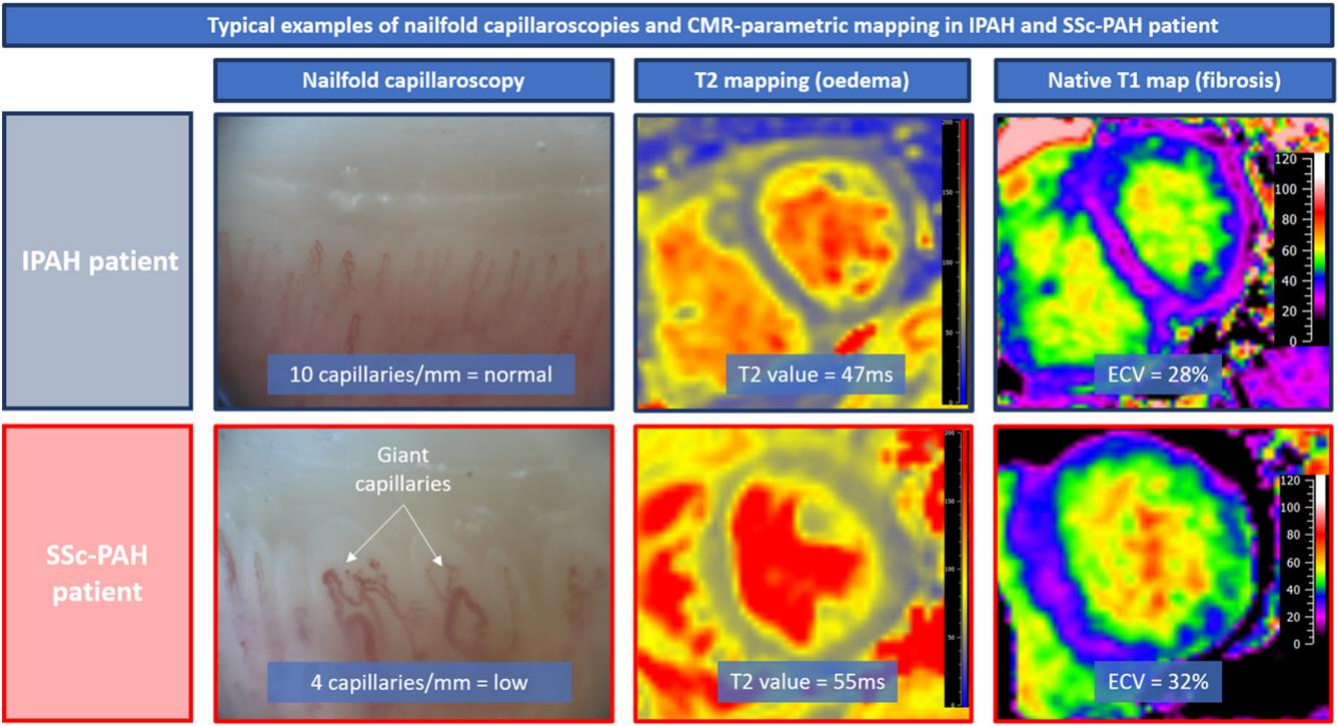
The figure shows a typical example of a picture of the nailfold capillaroscopy and parametric mapping in a patient with IPAH (in blue) and in a patient with SSc–PAH (in red). Most patients with SSc–PAH had a typical late SSc pattern on nailfold capillaroscopy, with low nailfold capillary density, whereas patients with IPAH had a normal or non-specific pattern with normal capillary density. In addition, patients with SSc–PAH had higher T_2_ values (cardiac inflammation) and a trend towards a higher ECV (cardiac fibrosis) compared with patients with IPAH. CMR, cardiovascular magnetic resonance; ECV, extracellular volume fraction; (I)PAH, (idiopathic) pulmonary arterial hypertension; SSc, systemic sclerosis.

Resting functional capillary density was lower in patients with SSc–PAH than in patients with IPAH, as demonstrated by a lower nailfold capillary density (NCD) and a lower rest capillary density at the PORH testing on the dorsal middle phalanx (*Table 4*). Additionally, patients with SSc–PAH had a lower average of capillaries during venous congestion (structurally available capillaries) and reduced recruitment of capillaries after PORH (a combination of functional and structural changes). *Figure 1* shows representative images of the NCM and parametric mapping in a patient with IPAH and a patient with SSc–PAH.

**Table 4:** Nailfold capillaroscopy with PORH testing

	SSc–PH (*n* = 20)	IPAH (*n* = 5)	*P*-value
Average capillary density (*n*/mm)	4 (3–6)	10 (10–11)	**<0.001**
Pattern (*n*)			
Normal	0	4 (80%)	
Non-specific	0	1 (20%)	
Systemic sclerosis: early pattern	1 (5%)	0	
Systemic sclerosis: active pattern	1 (5%)	0	
Systemic sclerosis: late pattern	18 (90%)	0	
PORH testing			
Average capillaries in rest (*n*/mm^2^)	55 (39–73)	93 (80–97)	**0**.**006**
Average capillaries after PORH (*n*/mm^2^)	53 (46–77)	100 (81–120)	**0**.**006**
Total recruitment of capillaries (*n*/mm^2^)^a^	1 (−4 to 5)	23 (−3 to 27)	**0**.**010**
Average capillaries at venous congestion (*n*/mm^2^)	55 (50–78)	106 (81–126)	**0**.**006**
Total recruitment of capillaries (*n*/mm^2^)^a^	5 (1–11)	11 (−9 to 30)	0.613

Values are in medians (interquartile range) or number (%). The *P-*values in bold indicate statistically significant correlations (*P* < 0.05). Abbreviations as in previous tables.

PORH, post-occlusive reactivity hyperaemia test.

^a^Number of extra capillaries recruited (minus the capillaries present in rest).

To assess whether the peripheral microvasculature mirrors a similar pattern of cardiac vasculopathy, reflected by myocardial inflammation or fibrosis, we correlated the indices of peripheral microvascular anatomy and function with the CMR tissue characteristics. T_2_ values and ECV negatively correlate with NCD (*Table 5*). In addition, the relative myocardial upslope (coronary microvascular function) correlates with both the number of capillaries during venous congestion and the number of capillaries after PORH.

**Table 5:** Correlation of the peripheral microvasculature on nailfold capillaroscopy with cardiac tissue characteristics and function

	Average nailfold capillary density (*n*/mm)	Capillaries at PORH (*n*/mm^2^)	Capillaries at venous congestion (*n*/mm^2^)
CMR tissue characterization and coronary microvascular function			
ECV (%)	**−0**.**443**	0.005	−0.093
T_2_ (ms)	−**0**.**464**	−0.109	−0.045
Relative myocardial upslope (%)	0.223	**0**.**421**	**0**.**467**
Diastolic functional indices and disease severity markers			
Indexed LV mass (g/m^2^)	**−0**.**448**	−0.395	**−0**.**415**
LA reservoir strain (%)	**0**.**475**	0.217	0.336
*E*ʹ lateral velocity (m/s)	**0**.**613**	0.263	0.311
*E*/*e*ʹ ratio	**−0**.**429**	−0.224	−0.254
Estimated RVSP (mmHg)	0.244	0.087	0.023
NT-proBNP (pg/mL)	**−0**.**460**	−0.407	**−0.437**
6MWD of predicted (%)	**0**.**439**	0.237	0.312
DLCO of predicted (%)	**0**.**575**	0.236	0.337

The *P*-values in bold indicate statistically significant correlations (*P* < 0.05). Abbreviations as in previous tables.

**Figure 2:**
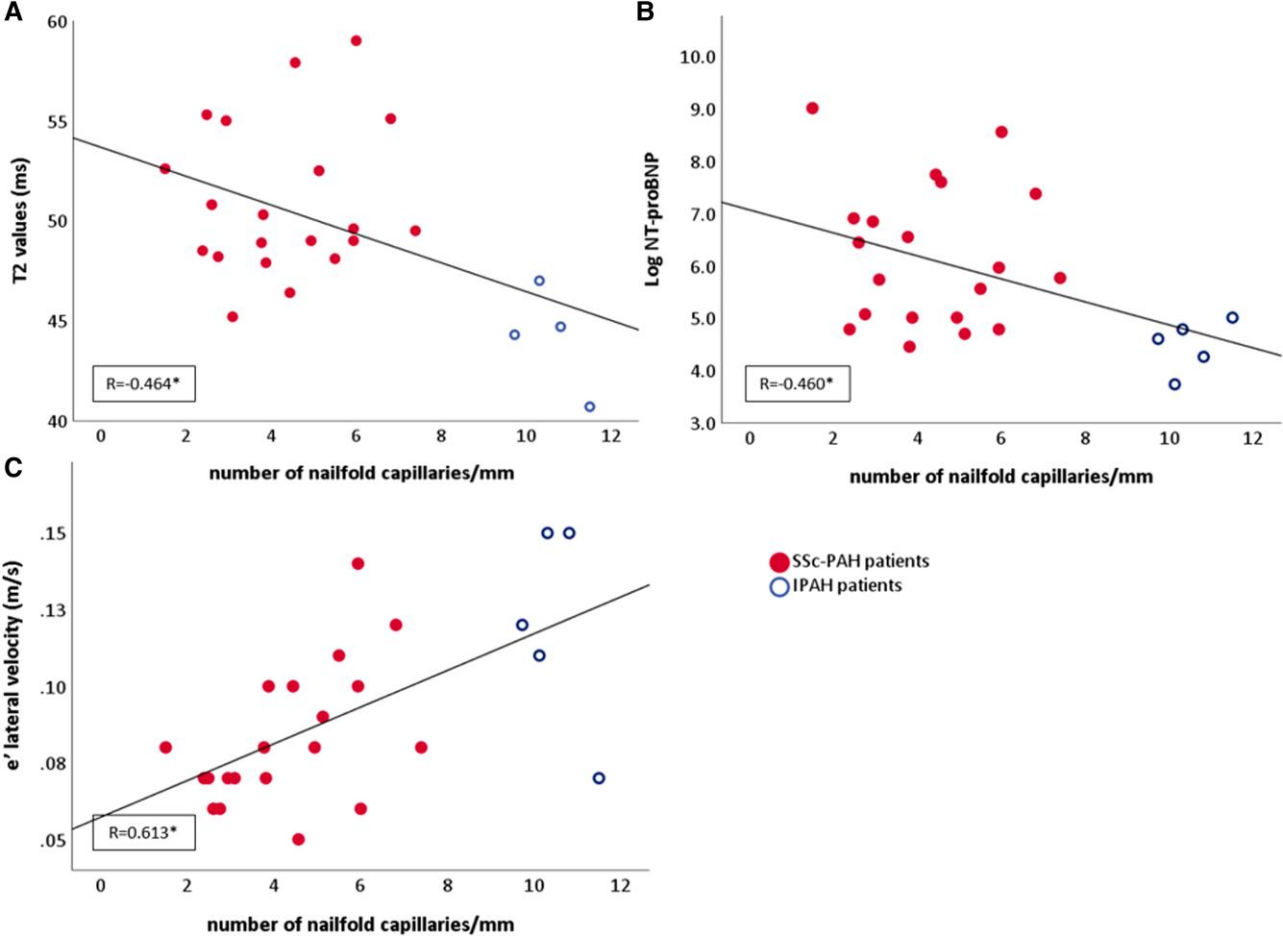
Scatter-dot graphs of the significant correlations between the NCD and the T_2_ values, *e*ʹ lateral velocity, and NT-proBNP. The average NCD correlates with T_2_ values (cardiac oedema) on cardiac magnetic resonance imaging (*A*), NT-proBNP levels (*B*), and *e*ʹ lateral velocity (*C*) on echocardiography. *P ≤ 0.05.

Finally, we evaluated the relationship between the peripheral microvasculature (structure and function) and ventricular and atrial function, to see whether the increase in myocardial oedema or fibrosis also translated into a decrease in cardiac function. On echocardiography, *e*ʹ lateral velocity and *E*/*e*ʹ ratios negatively correlate with NCD (*Table 5*). On CMR, both the indexed LV mass and the LA reservoir strain negatively correlate with NCD. In addition, NT-proBNP levels negatively correlate with lower NCD and lower capillaries at venous congestion, and DLCO of predicted was positively correlated with NCD. *Figure 2* shows the scatter plots of T_2_ values, *e*ʹ lateral velocities and NT-proBNP with the NCD. To note, there were no correlations with LV systolic function or RV volumes/function (data not provided).

## Discussion

Cardiac involvement and peripheral microvascular function in patients with SSc–PAH were evaluated and compared with patients with IPAH by undergoing an extensive 1-day study protocol, including CMR (stress) imaging, echocardiography, blood sampling, and NCM. The main findings are (i) patients with SSc–PAH showed diffuse cardiac involvement seen by higher T_2_ values and a trend towards a higher ECV, and worse peripheral microvascular function, compared with patients with IPAH and (ii) worse peripheral microvasculopathy correlates with diffuse cardiac involvement and microvascular coronary perfusion, and with worse diastolic LV function (*Graphical Abstract*). The correlations between peripheral microvascular function and markers of cardiac involvement suggest a common pathophysiological pathway of vasculopathy, inflammation, and fibrosis, and might contribute to the disproportionate high mortality in patients with SSc–PAH.

RV function is most important in patients with PAH, as the prognosis is not solely determined by the severity of the PAH, but to a great extent on the ability of the RV to adapt to the increased RV afterload.^3^ Patients with SSc–PAH are known to have a significantly worse prognosis than patients with IPAH,^2^ which is not attributable to more severe PAH or lower pulmonary arterial compliance.^4^ RV failure occurs earlier in the course of the disease, due to intrinsic myocardial dysfunction, seen by reduced contractile reserve and systolic function, and depressed sarcomere function.^4,24^ It is likely that primary myocardial involvement of SSc plays a pivotal role in the depressed cardiac function. Especially since, even without PAH, cardiac complications are common in SSc, and one of the leading causes of death.^25^ In our study, patients with SSc–PAH had higher T_2_ (oedema) and a trend towards higher ECV (fibrosis), implicating signs of diffuse cardiac involvement. The elevated T_2_ values implicate an increased cardiac inflammatory state in patients with SSc. Previous CMR studies of patients with SSc are in line with our results, showing diffuse and focal cardiac fibrosis, and signs of subclinical myocardial inflammation,^5,7^ even fulfilling the original and updated Lake Louis Criteria for diagnosing myocardial inflammation.^8,26^ SSc patients with silent myocarditis on CMR had improved CMR markers of inflammation at follow-up as a response on immunosuppressive therapy,^8^ suggesting that active myocardial inflammation might give a possible therapeutic target to prevent further cardiac deterioration. CMR might help detecting different cardiac phenotypes in SSc. A recent study of Knight *et al*.^20^ identified five CMR-based cardiac phenotypes associated with varying all-cause mortality rates. In our study, 45% of patients with SSc–PAH fell into the ‘RV failure’ or ‘normal function, large cavity’ group, and these patients had significantly higher ECV. In the study of Knight *et al.*, these clusters were associated with a worse prognosis compared with the normal function and average or small cavity groups. Early detection of SSc–PAH is associated with improved survival, and the development of evidence-based algorithms such as DETECT in order to diagnose PAH in SSc at an early stage are thought to have contributed to improved prognosis.^27^ Whether early intervention in patients with SSc with a specific CMR-based cardiac phenotype or myocardial inflammation on CMR improves outcome on the long term, still needs further investigation.

Patients with SSc–PAH had lower NCD and worse peripheral functional tests at venous congestion and at PORH, compared with patients with IPAH. This was expected, since SSc is known to severely affect the nailfold capillaries, and specific abnormalities are used as a classification criterion.^1,9^ A novel finding is that this peripheral microvascular dysfunction was also associated with higher T_2_ values and ECV, and a lower myocardial perfusion reserve on CMR. This suggests a common systemic driven inflammation, leading to fibrosis and microvasculopathy, also affecting the myocardium. The elevated inflammatory state and hence cardiac involvement importantly worsens prognosis in patients with SSc–PAH.^2^ Medication targeting systemic endothelial dysfunction could therefore be a promising target, both preventing pulmonary vascular remodelling as well as coronary microvascular dysfunction, to preserve RV function. Remarkably, there were no differences in myocardial perfusion measures between patients with SSc–PAH and IPAH. Compared with healthy controls, patients with SSc did have significantly decreased global myocardial perfusion.^16^ The sensitivity to detect subtle differences in the degree of microvascular dysfunction may be limited by the semiquantitative approach used in our study, particularly when considering the relatively small sample size. Another possible explanation could be that patients with IPAH also show a degree of coronary microvascular dysfunction, as reported previously.^28^ Another interesting finding is that there was an association between peripheral microvascular function and diastolic dysfunction on echocardiography and CMR, and also with semiquantitative measures of cardiac function (i.e. 6MWD, DLCO, and NT-proBNP levels). Future studies are needed to see if systemic microvascular dysfunction, via the pathophysiological pathway of endothelial dysfunction (and subsequent myocardial ischaemia) and the pro-inflammatory state, contributes to diastolic dysfunction in SSc.

### Limitations

The limited sample size in this pilot study is acknowledged, which may lead to false-positive findings (Type 1 errors) or to insufficient statistical power to detect significant differences (Type 2 errors), despite potential clinical relevance. Our results need further validation in a larger study population to see whether these findings are consistent and reproducible. Although LGE imaging and parametric mapping on CMR are the non-invasive gold standard for characterizing cardiac tissue, histopathological data are lacking. The severity of PAH could only be estimated by the RVSP on echocardiography, and the severity of ILD by the pulmonary function tests, since a right heart catheterization or a computed tomography scan was repeated only when clinically indicated. Whether the peripheral microvascular functional measurements or cardiac involvement on CMR can be used as markers to initiate early, patient-tailored therapy needs to be investigated in future studies.

## Conclusion

Patients with SSc–PAH showed signs of diffuse cardiac involvement on CMR and worse peripheral microvascular function compared with patients with IPAH. In addition, peripheral microvascular function correlates with cardiac fibrosis, inflammation, and myocardial perfusion on CMR. The correlations between peripheral microvascular function, cardiac fibrosis, and inflammation, and measures of diastolic dysfunction suggest a common link and may explain why patients with SSc–PAH experience cardiac failure earlier in the course of the disease. Our findings indicate that SSc causes a more severe inflammation, fibrosis, and vasculopathy of both the heart and the peripheral microvasculature. Our results stress the need for a larger prospective study, preferably multicentre, to assess whether myocardial involvement in patients with SSc can serve as a prognostic indicator or guide early interventions such as anti-inflammatory/anti-fibrotic therapy, to improve outcome.

## Supplementary data

Supplementary data are available at *European Heart Journal - Cardiovascular Imaging* online.

## Supplementary Material

jeae001_Supplementary_Data

## Data Availability

The data that support these findings are available on reasonable request to the corresponding author.
