# Supplementary material for: Peripheral microvascular function is linked to cardiac involvement on cardiovascular magnetic resonance in systemic sclerosis–related pulmonary arterial hypertension
Source: Eur Heart J Cardiovasc Imaging. 2024 Jan 3;25(5):708–17. doi: 10.1093/ehjci/jeae001 (PMC11057940; doi:10.1093/ehjci/jeae001)
Supplement: jeae001_Supplementary_Data [file jeae001_supplementary_data.docx]

**SUPPLEMENTAL METHODS**

**Echocardiography acquisition**

The TTE was performed with a Toshiba Artida system (Toshiba) with a 5-MHz transducer, or a GE medical system VIVID E9 (GE Healthcare) with a 1.5-4MHz phased array transducer. Offline analysis was performed on commercially available software. Using pulsed wave Doppler-imaging, the diastolic function was assessed by measuring mitral inflow, including the peak early filling (E-wave) and late diastolic filling (A-wave) velocities, the E-wave deceleration time, and the early diastolic velocities at the lateral mitral annulus (e’ lateral). E/A ratios and E/e’ lateral ratios were calculated. RV systolic pressure (RVSP) was calculated by adding the Bernoulli equation derived pressure gradient from the maximum tricuspid regurgitation velocity to the estimated right atrial (RA). Tricuspid annular plane systolic excursion (TAPSE) was measured on the apical 4-chamber view using M-mode echocardiography.

**Peripheral microvascular evaluation**

The nailfold capillaries were imaged using an optical probe video capillaroscope with a 200x contact lens and connected to analysis software (Optilia OP-120 011, Mediscope Digital, videomicroscope (USB interface), OptiPix Capillaroscopy software, clinic 1.7.x with a 200x high resolution objective lens with unpolarized light). For the quantitative and qualitative analysis, 2 images per finger were evaluated.

PORH testing was performed in a quiet room with a temperature around 22°C, and the patient positioned in a supine position. The images were taken from the dorsal skin of the middle phalanx of the third finger of the non-dominant hand. Images were acquired in rest, directly after arterial occlusion (achieved by inflating a blood pressure cuff >60mmHg above systolic pressure for 3 minutes), and during venous congestion (achieved by inflating the blood pressure cuff to a pressure of 60mmHg for 2 minutes) [1, 2]. All capillaries within 1mm^2^ were counted (4 images per phase) by two investigators (JV and JL). If there was a discrepancy of the number of capillaries >10% between investigators, the images were recounted. The average number of capillaries/mm^2^ of the 4 images were used for further analysis. The capillaries at rest represent the functional capillary density. The capillaries at venous congestion represent the structural number of capillaries, including the non-perfused capillaries (not visible in rest). Temporary arterial occlusion creates reactive hyperemia by the release of endothelial mediators resulting in vasodilation, and therefore total number of capillaries after PORH represent both functional and structural changes [1, 2].

**CMR acquisition**

All patients were scanned on a commercially available clinical CMR scanner (1.5 Tesla Siemens Avanto), using a phased array cardiac receiver coil. Balanced steady-state free-precession (long- and short-axis) cine images were acquired during end-expiratory breath holds, with the following typical sequence parameters: TR/TE 3.4/1.69ms, voxel size 1.3 x 1.3 x 8.0mm, flip angle of 55°, and 30 phases per cardiac cycle. The consecutive short-axis cine images from base to apex were used to analyze RV and LV mass, volumes, and calculate ejection fraction (EF). Parametric mapping was performed on the 4-chamber long-axis and basal, mid, and apical short-axis views. T2 mapping was acquired using a bright-blood T2 prepared, steady-state free precession sequence.[3] Native and post-contrast T1 mapping images were acquired using the Shortened Modified Look-Locker Inversion Recovery sequence. Hematocrit, to calculate the extracellular volume fraction (ECV), was measured on the same day as the CMR acquisition.[3] Stress T1 maps on three short axis levels (basal, mid and apical) were acquired 3 minutes after continuous infusion of adenosine to evaluate microvascular coronary function [4]. Immediately after the stress T1 maps, stress first pass perfusion imaging was performed with a gadolinium-based contrast agent (Dotarem, Guerbet, Rossy, France; 0.1 mmol/kg at a rate of 4.5ml/s) to measure the relative myocardial upslope, and to evaluate regional perfusion defects to exclude significant epicardial coronary stenoses. Ten to 15 minutes after contrast injection, LGE images were acquired using a two-dimensional, segmented inversion-recovery prepared gradient echo pulse sequence in similar views as the short- and long-axis cine images.

**CMR analysis**

All post-processing analyses were performed using Medis Qstrain software (Medis Medical Imaging Systems, version 2.0.48.8, the Netherlands). LV- and RV volumes and mass were measured on the short-axis cine images, and the ejection fraction (EF) was calculated. Left atrial (LA) and right atrial (RA) volumes were calculated using the biplane Simpson’s area-length method. The presence of LGE was visually evaluated and assessed as present if it was visible on the same location in 2 different views (short- and long-axis).

*Feature tracking strain:* LA strain was the average strain measured on the 2- and 4-chamber long axis cine images, LV global longitudinal strain (GLS) as the average of the 2-, 3-, 4-chamber long axis cine images. RA strain and RV GLS were measured on the 4-chamber long axis cines. The endocardial contours are manually drawn in the end-systolic and end-diastolic phase, the software automatically tracks the contours in the consecutive frames. LA and RA phasic strain parameters include reservoir (expansion of the atria filling with blood), conduit (passive filling of blood from the atrium to the ventricle), and booster strain (atrial contraction).

*Parametric mapping:* The analysis was performed in accordance with the recommendations of the Society of Cardiovascular Magnetic Resonance, wherein diffuse cardiac disease was assessed by manually delineating a single region of interest on the raw images of the native and post-contrast T1 maps and T2 maps, using the septal mid-ventricular short-axis images.[5] Additionally, the entire myocardium (excluding regions with focal LGE) was delineated using endo- and epicardial contours on all three short-axis and 4-chamber long-axis images.[5] Areas of LGE were excluded from analysis. The extracellular volume fraction was used to evaluate cardiac fibrosis. T2-mapping was used to evaluate cardiac oedema. T2 ≥55ms was defined as a sign of myocardial oedema. A region in the septum was manually drawn on the automatically generated T2 maps of the mid-slice short-axis T2 images.

*Microvascular coronary perfusion:* This was evaluated by two different methods: measuring the relative myocardial upslope and by measuring the myocardial reactivity on adenosine stress using stress T1 mapping. The semiquantitative analysis of myocardial perfusion during stress was calculated by measuring the mean relative myocardial upslope of the signal intensity time curve of the contrast agent. LV endo- and epicardial contours were drawn on the 3 short-axis first-pass perfusion images, as described previously,[6] and the mean relative myocardial upslope was automatically calculated. Using the T1 maps, the mean myocardial reactivity on adenosine stress was measured (ΔT) using the following formula: (stress T1 – native T1)/native T1*100.[4]

| **Supplemental Table S1. Parametric mapping analysis** | | | |
| --- | --- | --- | --- |
|  | SSc-PH (n=20) | IPAH (n=5) | p-value |
| Septal mid-ventricular native T1 value (ms) | 966 [932-985] | 899 [891-921] | 0.004 |
| Overall native T1 value (ms)* | 960 [932-978] | 917 [896-928] | 0.006 |
| Septal mid-ventricular ECV (%) | 30 [28-34] | 28 [24-29] | 0.060 |
| Overall ECV (%) | 31 [30-35] | 28 [27-30] | 0.015 |
| Septal mid-ventricular T2 values (ms)† | 50 [48-54] | 45 [42-46] | 0.001 |
| Overall T2 values (ms) | 52 [51-54] | 47 [45-49] | <0.001 |
| *Abbreviations:* (I)PAH, idiopathic pulmonary arterial hypertension; SSc, systemic sclerosis.  * Average value of the entire myocardium, using endo- and epicardial contours on all three short-axis and 4-chamber long-axis; † T2 map missing in one IPAH patient.  T1 values and extracellular volume fractions are measured outside areas of LGE. | | | |

**REFERENCES**

1. Serne, E.H., et al., *Impaired skin capillary recruitment in essential hypertension is caused by both functional and structural capillary rarefaction.* Hypertension, 2001. **38**(2): p. 238-42.

2. Tibirica, E., et al., *Reduced systemic microvascular density and reactivity in individuals with early onset coronary artery disease.* Microvasc Res, 2015. **97**: p. 105-8.

3. Messroghli, D.R., et al., *Clinical recommendations for cardiovascular magnetic resonance mapping of T1, T2, T2* and extracellular volume: A consensus statement by the Society for Cardiovascular Magnetic Resonance (SCMR) endorsed by the European Association for Cardiovascular Imaging (EACVI).* J Cardiovasc Magn Reson, 2017. **19**(1): p. 75.

4. Liu, A., et al., *Adenosine Stress and Rest T1 Mapping Can Differentiate Between Ischemic, Infarcted, Remote, and Normal Myocardium Without the Need for Gadolinium Contrast Agents.* JACC Cardiovasc Imaging, 2016. **9**(1): p. 27-36.

5. Schulz-Menger, J., et al., *Standardized image interpretation and post-processing in cardiovascular magnetic resonance - 2020 update : Society for Cardiovascular Magnetic Resonance (SCMR): Board of Trustees Task Force on Standardized Post-Processing.* J Cardiovasc Magn Reson, 2020. **22**(1): p. 19.

6. Everaars, H., et al., *Cardiac Magnetic Resonance for Evaluating Nonculprit Lesions After Myocardial Infarction: Comparison With Fractional Flow Reserve.* JACC Cardiovasc Imaging, 2020. **13**(3): p. 715-728.
